# Infrared reflectance image-guided laser photocoagulation of telangiectatic capillaries in persistent diabetic macular edema

**DOI:** 10.1038/s41598-021-01183-9

**Published:** 2021-11-05

**Authors:** Hyeon Cheol Roh, Chaeyeon Lee, Se Woong Kang, Kyung Jun Choi, Jun Soo Eun, Sungsoon Hwang

**Affiliations:** 1 Department of Ophthalmology, Samsung Changwon Hospital, Sungkyunkwan University School of Medicine, Changwon, Korea; 2 Department of Ophthalmology, Samsung Medical Center, Sungkyunkwan University School of Medicine, Seoul, Korea

**Keywords:** Retinal diseases, Vision disorders

## Abstract

This study aimed to assess detection rate of telangiectatic capillaries (TelCaps) with infrared reflectance (IR) and optical coherence tomography (OCT) images and to evaluate the clinical efficacy of IR image-guided focal laser photocoagulation of TelCaps in persistent diabetic macular edema (DME). This retrospective case series included 28 eyes of 28 patients with TelCap and persistent DME refractory to intravitreal anti-vascular endothelial growth factor or corticosteroids. The presence of TelCaps was confirmed using IR and OCT images. All patients were followed up for more than 12 months after direct focal laser photocoagulation of the TelCaps. The TelCap closure rate, changes in best-corrected visual acuity, and central subfield thickness were analyzed. On IR imaging, TelCap appeared as a characteristic hyperreflectivity within a hyporeflective spherical lesion in 85.7% of the eyes. After focal laser photocoagulation, the TelCap closure rate was 57.1% at 3 months and 71.4% at 12 months. A significant improvement in visual acuity and reduction in central subfield thickness were observed at three and 12 months after focal laser photocoagulation (all p < 0.05). The characteristic hyperreflectivity within hyporeflective lesions on the IR image in conjunction with OCT helps identify the TelCap. Our results suggest that IR imaging and OCT-guided focal laser photocoagulation of TelCaps can improve functional and anatomical outcomes in persistent DME.

## Introduction

Diabetic macular edema (DME) is a common cause of moderate vision loss among patients with diabetic retinopathy^1^. For decades, focal and grid macular laser photocoagulation has been the standard treatment for DME^1^. In recent years, non-surgical treatments, including intravitreal injection of anti-vascular endothelial growth factor (VEGF) and periocular/intravitreal corticosteroid injection, have contributed to the significant improvement in visual outcome for patients with DME. In particular, anti-VEGF therapy has shown superior efficacy for treating DME in several large randomized clinical trials^2–5^, and has become the first-line treatment for DME. However, macular edema does not improve, but rather persists in a significant number of patients despite repeated intravitreal injections of anti-VEGF and/or corticosteroid^6–8^.

Microvascular abnormalities such as microaneurysms are the most characteristic sign of diabetic retinopathy, and extravascular leakage from these can cause macular edema^9^. Occasionally, microvascular abnormalities reach several hundreds of microns in diameter, and such large lesions have been termed capillary macroaneurysms^10,11^. A pilot study reported that leakage from capillary macroaneurysms was associated with persistent DME refractory to intravitreal treatment^10^. Castro-Farías et al. recently proposed the name ‘telangiectatic capillaries’ (TelCaps) to describe capillary abnormalities larger than 150 μm to avoid confusion with the term ‘retinal arterial macroaneurysm’^12^.

Indocyanine green angiography (ICGA) is known to be useful for the detection of TelCaps^10,11^. Several studies have reported that ICGA-guided direct focal laser photocoagulation for TelCaps improves the visual and anatomical outcomes of DME^10,13^. However, in real-world clinical practice, performing ICGA in all persistent DME patients can be difficult because of its invasive and time-consuming nature. In contrast, optical coherence tomography (OCT) is a non-invasive, rapid imaging modality for quantitative retinal thickness measurements, which is essential for the detection and monitoring of DME^14^. In addition, confocal infrared reflectance (IR) images can be simultaneously acquired during OCT imaging.

This study aimed to assess detection rate of TelCaps with IR and OCT images and to evaluate the clinical efficacy of IR image-guided focal laser photocoagulation of TelCaps in persistent DME.

## Methods

We reviewed the medical records of 28 patients who underwent direct focal laser photocoagulation between March 2011 and July 2019 for the treatment of persistent DME associated with TelCaps. This study conformed to the tenets of the Declaration of Helsinki and was approved by the Institutional Review Board of the Samsung Medical Center (No. 2021-01-170), which waived the written informed consent because of the study’s retrospective design.

The eligibility criteria were as follows: (1) clinically significant macular edema that persisted for more than three months after initiation of successive intravitreal injections; (2) reduction of less than 10% in central subfield thickness despite intravitreal anti-VEGF or periocular/intravitreal corticosteroid injections; (3) presence of one or more TelCaps with a diameter of at least 150 µm determined by OCT with IR and a focal edema component associated with the TelCap; (4) more than 12 months of follow-up after focal laser photocoagulation; and (5) age > 20 years. Eyes with significant media opacity or coexisting macular diseases, including epiretinal membrane, vitreomacular traction, age-related macular degeneration, or retinal vascular obstruction, were excluded.

Assessment of TelCaps associated with macular edema was routinely performed using IR and OCT. Color fundus photograph shows that TelCap associated with blot retinal hemorrhage and, in some cases, hard exudates. OCT (SPECTRALIS, Heidelberg Engineering, Heidelberg, Germany) was performed using the scan protocol of 6 mm radial scans of 30° intervals, in addition to a horizontal raster scan that transected the center of each TelCaps. OCT showed that the TelCap had a round or ovoid structure, presenting a thick hyperreflective wall surrounding a hyporeflective lumen^10–12^. On the IR image, which was provided as default OCT imaging, the TelCap appeared as a hyporeflective spherical lesion with or without central hyperreflectivity. This imaging result is because blood is the main absorber in the near-infrared spectrum and therefore retinal vessels appear dark in IR images^15^. ICGA was occasionally used to help confirm the TelCap, showing early dye filling and increasing intensity of the lesion's hyperfluorescence with limited leakage in the late phase^11^. The diameter of TelCap and the distance between the center of the TelCap and fovea was also assessed using a built-in software on the OCT device.

A single retinal specialist (S.W.K.) performed the focal laser photocoagulation in all cases. The laser was only applied to the TelCap lesions and was not applied to the microaneurysms. Prior to laser treatment, the TelCaps were determined and localized using IR and OCT images. All treatments were performed under topical anesthesia with a fundus contact lens (AREA CENTRALIS; Volk Optical Inc., Mentor, OH, USA). Two patients required retrobulbar anesthesia to block excessive eye movement while applying laser photocoagulation to the Telcap lesion. The endpoint of focal laser photocoagulation was to produce a grayish-white burn at the center of the TelCap with the following laser parameters: argon green wavelength, the spot size of 50–70 μm, duration of 0.06–0.1 s, and power of 50–100 mW. The TelCap closure following laser photocoagulation was defined as the disappearance of a hyporeflective lumen on OCT^16^. We also assessed changes in the outer retina at the focal laser site on OCT scans through the fovea and the presence of retinal pigment epithelial degeneration on serial fundus examinations and IR images.

Baseline clinical characteristics, including the number of prior intravitreal anti-VEGF or periocular/intravitreal corticosteroid injections, the grade of diabetic retinopathy, and history of pan-retinal photocoagulation, were collected at enrollment. All patients underwent a complete ophthalmological examination, including best-corrected visual acuity (BCVA) measurement with manifested refraction, intraocular pressure, slit-lamp biomicroscopy, dilated funduscopy with a 90-diopter lens, fundus photography, spectral-domain OCT with IR imaging, and fluorescein angiography at baseline.

BCVA and central subfield thickness on OCT were assessed at baseline and at 3 and 12 months after laser treatment. BCVA was recorded using the Snellen chart and converted to the logarithm of the minimum angle of resolution for statistical analysis.

IBM SPSS ver. 25.0 (IBM Corp., Armonk, NY, USA) was used for all the statistical analyses. The paired *t*-test was performed to compare BCVA and central subfield thickness at baseline and after laser photocoagulation. P values less than 0.05 were considered statistically significant.

### Ethics approval

Institutional Review Board of Samsung Medical Center (No. 2021-01-170).

## Results

In total, 28 eyes of 28 patients who met the eligibility criteria were analyzed. All the patients had well-controlled type 2 diabetes mellitus, and their lipid profiles and systemic blood pressure were within acceptable ranges. None of the 28 eyes underwent cataract surgery during the study period. The patients' mean age was 66.5 ± 9.5 years, and 21.4% of the patients were female. Baseline clinical details are presented in Table 1. Prior to laser treatment, the mean number of intravitreal anti-VEGF injections was 2.64 ± 2.51, and the mean number of periocular/intravitreal corticosteroid injections was 1.32 ± 1.39. TelCaps associated with persistent DME were solitary in 92.9% of patients and multiple in 7.1% of the patients. The mean distance of TelCaps from the foveal center was 1299.5 ± 907.2 μm.

**Table 1:** Baseline characteristics of patients included in the study.

Characteristic	Data
Eyes, n	28
Age, years	66.5 ± 9.5
Sex, male:female	22:6
BCVA (logMAR)	0.55 ± 0.52
Central subfield thickness, μm	400.93 ± 147.14
Prior intravitreal anti-VEGF injections, n	2.64 ± 2.51
Prior periocular or intravitreal corticosteroid injections, n	1.32 ± 1.39
**Grade of diabetic retinopathy, n (%)**	
NPDR	17 (60.7)
PDR	11 (39.3)
PRP history, n (%)	15 (53.6)
**Number of TelCaps, n (%)**	
Solitary	26 (92.9)
Multiple	2 (7.1)
Distance from fovea, μm	1299.5 ± 907.2

Values are presented as mean ± standard deviation or percentages.

*BCVA* best-corrected visual acuity, *LogMAR* logarithm of the minimum angle of resolution, *VEGF* vascular endothelial growth factor, *NPDR* non-proliferative diabetic retinopathy, *PDR* proliferative diabetic retinopathy, *PRP* panretinal photocoagulation, *TelCap* telangiectatic capillary.

**Figure 1:**
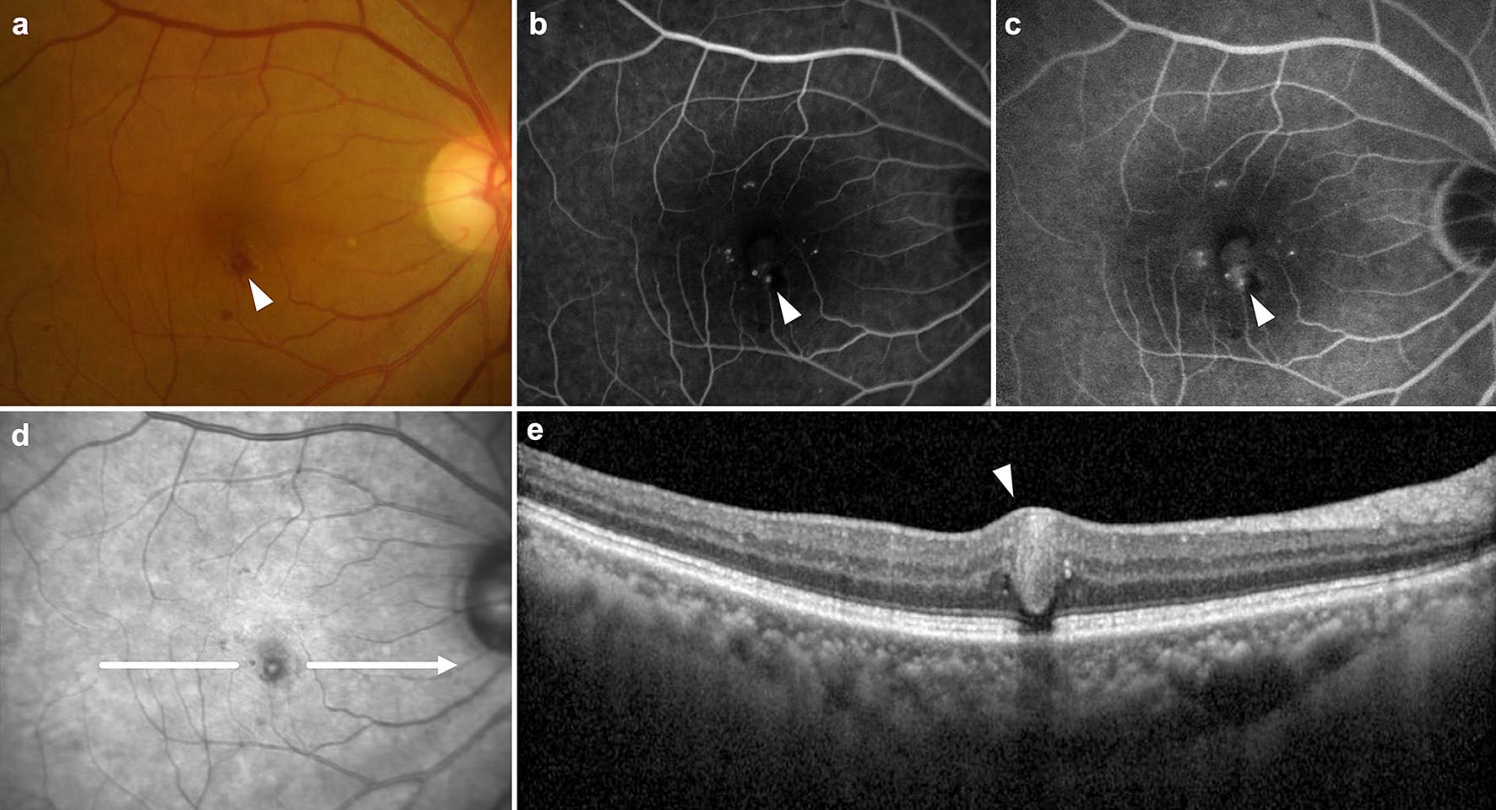
Multimodal imaging of a telangiectatic capillary (TelCap) (arrowhead) in persistent diabetic macular edema. (**a**) Color fundus photo shows intraretinal hemorrhages, microaneurysms, and TelCap with several hard exudates inferior to the fovea. The TelCap appears as a reddish round superficial lesion with a diameter larger than 150 µm. Fluorescein angiography shows multiple hyperfluorescent lesions including microaneurysms and TelCaps in the early phase (**b**) and leakage of TelCaps in the late phase (**c**). (**d**) An infrared reflectance image reveals that the TelCap is characterized by hyperreflectivity inside a hyporeflective spherical lesion, distinguishing it from other microaneurysms. (**e**) Optical coherence tomography shows a hyporeflective ovoid lesion surrounded by a hyperreflective TelCap wall.

Figure 1 shows a case of TelCap in persistent DME on multimodal imaging. In the IR image, central hyperreflectivity was observed within a hyporeflective spherical lesion. This characteristic finding on IR imaging was observed in 85.7% of patients.

**Figure 2:**
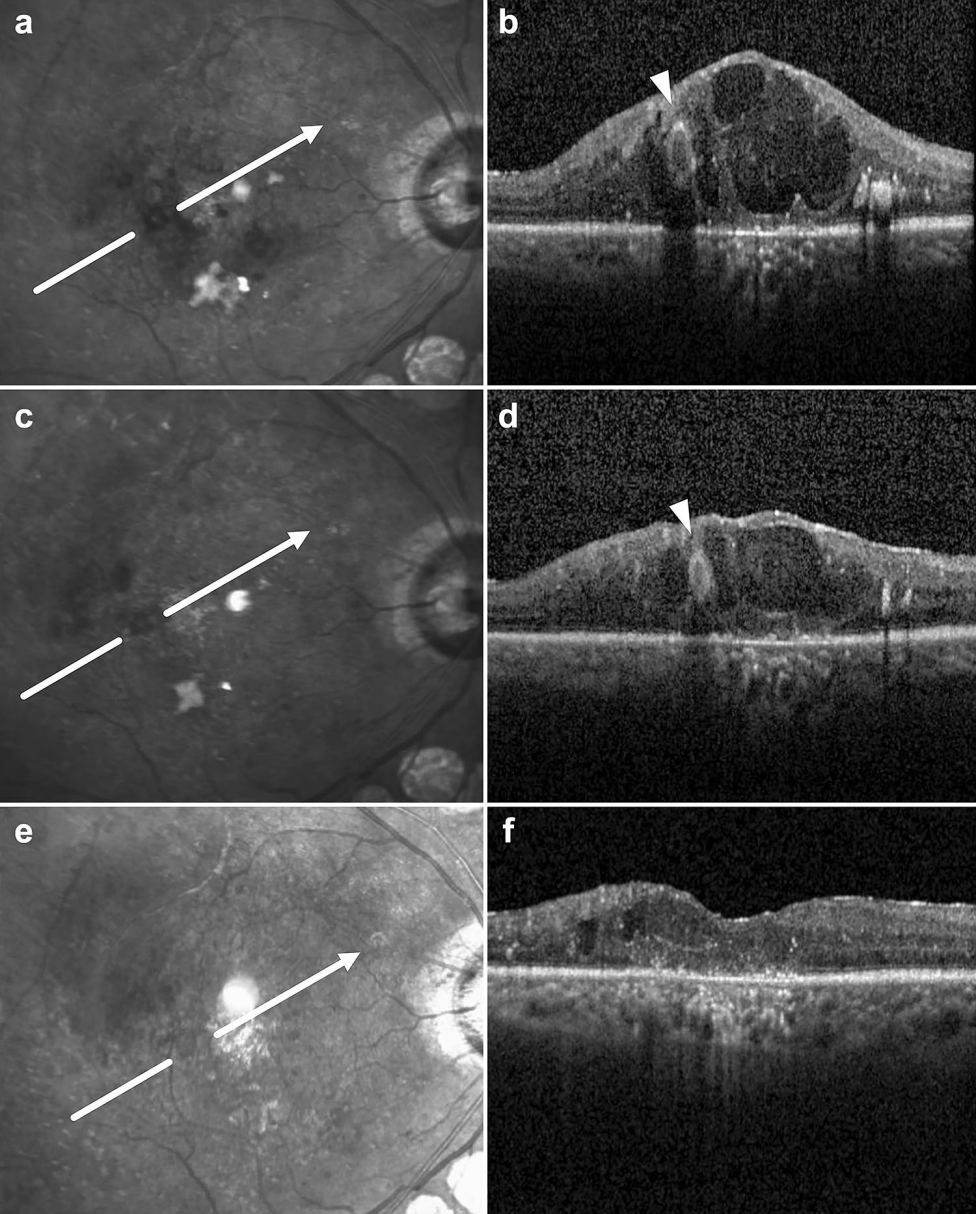
Infrared (IR) reflectance and spectral-domain optical coherence tomography (OCT) images of the telangiectatic capillary (TelCap) (arrowhead) before (**a**,**b**) and after (**c**–**f**) focal laser photocoagulation in persistent diabetic macular edema. (**c**,**d**) Three months after focal laser photocoagulation of the TelCap, the TelCap size decreased on both the IR image and OCT, and the macula edema improved. (**e**,**f**) One year following laser treatment, the TelCap lesion disappeared, and the macula edema and lipid exudates around the TelCap were resolved markedly. The findings outlined above showed the relationships between TelCap lesion and these macular changes.

**Figure 3:**
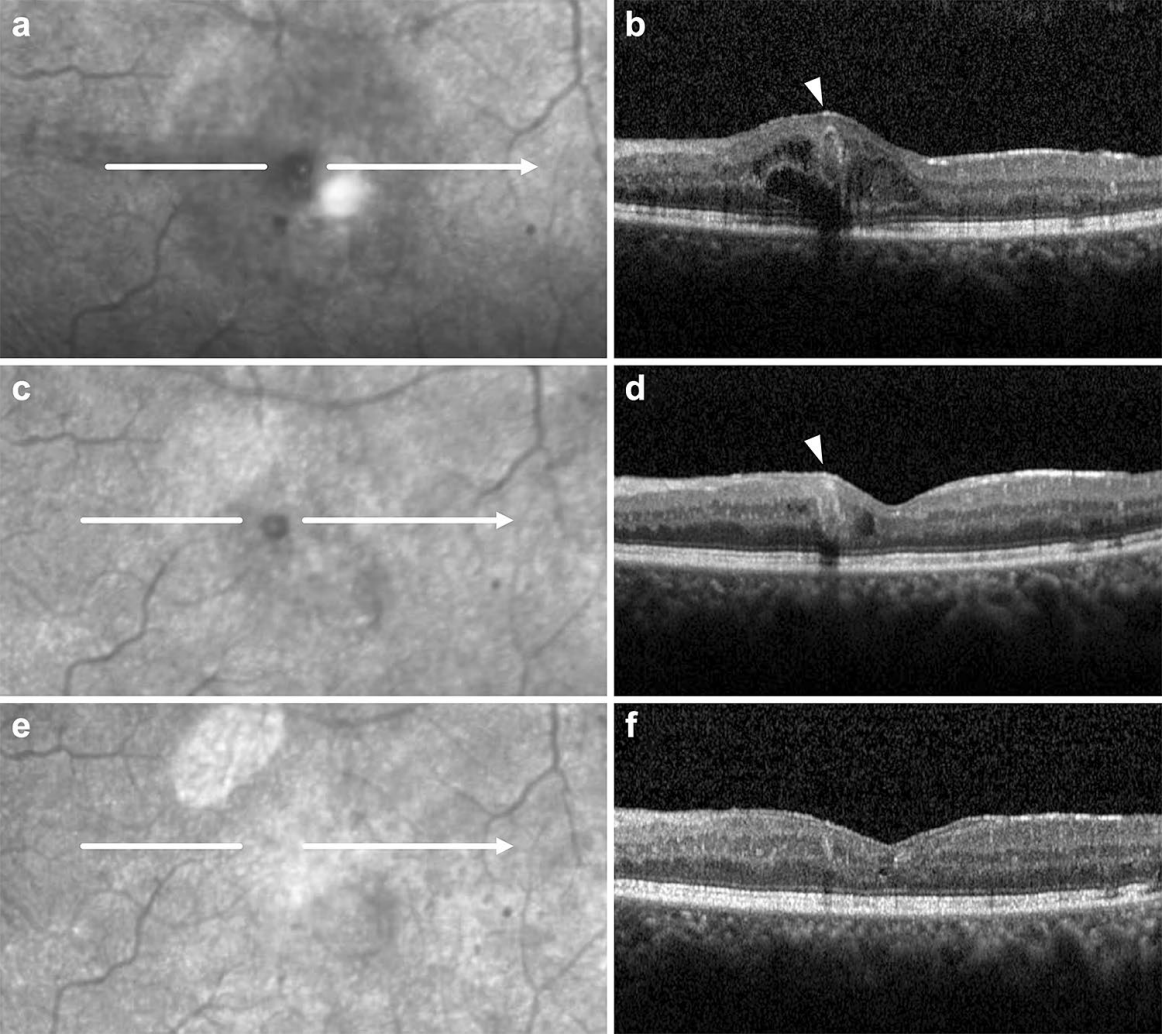
Magnified infrared (IR) reflectance and spectral-domain optical coherence tomography (OCT) scans of the telangiectatic capillary (TelCap) (arrowhead) before (**a**,**b**) and after (**c**–**f**) focal laser photocoagulation in persistent diabetic macular edema. As the size of the TelCap on OCT decreased after focal laser photocoagulation (**d**), the size of hyporeflective lesion on the IR image also decreased (**c**), and eventually disappeared (**e**) with complete closure of the TelCap (**f**).

After laser treatment, the TelCap closure rate was 57.1% at 3 months and 71.4% at 12 months. Figure 2 shows an example of TelCap closure following focal laser photocoagulation on OCT and IR images. As the size of TelCaps on OCT decreased after focal laser photocoagulation, the size of the hyporeflective lesion on the IR image also decreased. In patients with complete TelCap closure on OCT following laser treatment, the hyporeflective lesion on the IR image also disappeared (Fig. 3). Eight patients (28.6%) who did not achieve complete TelCap closure during the study period were reviewed after 12 months of follow-up. Among them, three patients showed spontaneous resolution of macular edema over time (2–5 years), and two patients showed no significant deterioration of visual acuity and macular edema after the 12-month follow-up and required no additional treatments. One patient with aggravation of DME underwent vitrectomy, and another patient experienced fresh vitreous hemorrhage with active neovascularization and was treated with intravitreal injections of bevacizumab. In addition, one patient was lost to follow-up after 12 months.

**Figure 4:**
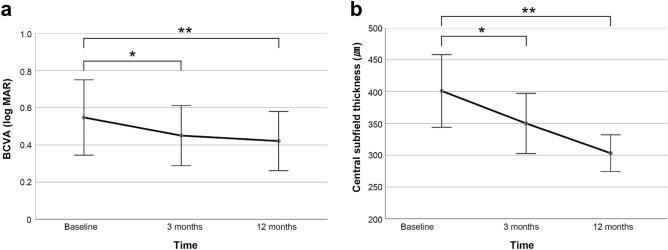
Changes in best-corrected visual acuity (BCVA) (**a**) and central subfield thickness (**b**) after focal laser photocoagulation in patients with a telangiectatic capillary in persistent diabetic macular edema. Error bars denote 95% confidence intervals. Asterisks indicate a statistically significant difference compared to baseline (*p < 0.05, **p < 0.005).

Changes in BCVA and central subfield thickness are shown in Fig. 4. The mean BCVA at baseline was 0.55 ± 0.52. After laser treatment, mean BCVA was significantly improved from baseline to 0.45 ± 0.42 at three months, and 0.42 ± 0.41 at 12 months (p = 0.018, p = 0.002, respectively). The mean central subfield thickness significantly decreased from 400.9 ± 147.1 μm at baseline to 350.1 ± 121.6 μm at 3 months, and to 303.2 ± 74.5 μm at 12 months after laser treatment (p = 0.009, p = 0.002, respectively).

Most patients did not undergo intravitreal treatment after focal laser photocoagulation to TelCaps. However, rescue anti-VEGF injections were applied in two of 28 patients who experienced aggravation of macular edema after focal laser treatment. These two patients did not demonstrate improvement in visual acuity or central subfield thickness at one year compared to baseline.

Following laser photocoagulation, retinal pigment epithelial changes affecting the fovea were not observed in any of the cases. Moreover, there were no accidental laser burns of the fovea in any of the cases.

## Discussion

The majority of telangiectatic capillaries could be diagnosed using OCT and IR images in our case series. Second, this study also suggested that IR imaging and OCT-guided focal laser photocoagulation of TelCap could achieve anatomical and functional improvement in refractory DME related to TelCap.

Persistent DME refractory to various non-surgical treatments can be challenging to treat and manage^17,18^. A previous study reported that TelCap was associated with chronic macular edema refractory to intravitreal treatment^10^. ICGA is known to be useful in the diagnosis of TelCap, showing delayed staining with limited leakage in the late phase^12^. However, the clinical use of ICGA could be challenging in real-world practice because it is invasive, costly, and time-consuming. Bourhis et al. reported that OCT is highly sensitive for the detection of TelCap and superior to ICGA because of the visualization of the TelCap wall^11^. On OCT image, TelCap was characterized by a round or ovoid structure with a thick hyperreflective wall surrounding a hyporeflective lumen, which was particularly useful for the diagnosis of TelCap.

A system combining OCT with a confocal scanning laser ophthalmoscope allows for simultaneous cross-sectional OCT with IR images and various topographic images^19^. IR imaging uses a near-infrared light source (wavelength of 820 nm) and assesses the various absorption, reflection, and scattering properties of ocular fundus tissues, determining the structural changes of the retina and choroid. Oxygenated and deoxygenated hemoglobin is a significant absorber of light in the near-infrared spectrum and, consequently, retinal vessels are seen as dark structures in IR images. TelCap, one of the microvascular abnormalities of diabetic retinopathy, also appears as a hyporeflective spherical lesion in IR images. An interesting finding of this study is that most TelCaps showed characteristic hyperreflectivity within hyporeflective lesions on IR images. The histological correlates of this hyperreflectivity are uncertain. Stitt et al. reported that intraluminal clots with blood cells and lipid deposits were histologically observed within microaneurysms^20^. They also described the intraluminal clotting with the extracellular matrix and lipid-containing macrophages within microaneurysms^20^. Castro-Farías et al. suggested that hydrophobic intraluminal materials in TelCap may induce incomplete and delayed filling in ICGA^12^. Hyperreflectivity in a blood vessel on IR imaging indicates a defect of red blood cell flow. Considering these observations, we hypothesize that the central hyperreflectivity of TelCaps on IR imaging is due to intraluminal hydrophobic deposits inside the TelCap.

Our results suggested that the characteristic hyperreflectivity within hyporeflective spherical lesions might be a distinguishing feature of TelCap from other hyporeflective lesions, such as microaneurysms or retinal hemorrhage on IR images. These characteristics can determine TelCap on IR images and improve targeting of focal laser treatment with the corresponding OCT images.

Furthermore, our results showed that as the TelCap size decreased on OCT following focal laser photocoagulation, the size of the hyporeflective lesion on the IR image also decreased. The hyporeflective lesion eventually disappeared when TelCap was completely closed (Figs. 2 and 3). Based on these findings, we suggest that an IR image with OCT may be useful for the follow-up evaluation of TelCap and its diagnosis.

The efficacy of focal laser photocoagulation for leaking microaneurysms has been proven in treating DME^1^. However, only a few pilot studies have examined the efficacy of focal laser photocoagulation of TelCap. Paques et al. evaluated the outcome of ICGA-guided targeted laser photocoagulation of TelCap in four eyes with DME and five eyes with retinal vein occlusion^10^. They reported a reduction in macular thickness and improvement of visual acuity 6 months after laser photocoagulation. Ogura et al. also reported the efficacy of ICGA-guided laser photocoagulation of TelCap in combination with a sub-tenon injection of triamcinolone acetonide in eight persistent DME patients^13^. The present study confirmed the significant improvement in BCVA and reduction in central subfield thickness at 3 and 12 months after IR and OCT-guided focal laser photocoagulation of TelCaps, through a relatively large number of cases. Our results also showed favorable TelCap closure rates after focal laser photocoagulation in persistent DME (57.1% at 3 months and 71.4% at 12 months). Regarding leaking microaneurysms in DME, Lee et al. reported that the closure rates evaluated by OCT were 70.0% at 3 months and 82.9% at 12 months after focal laser photocoagulation^16^, which were relatively higher than those of our results. This difference might be caused by the larger TelCap size compared to that of the microaneurysm, resulting in an extended period for complete closure. Indeed, it has been reported that a larger microaneurysm size is associated with a low closure rate after focal laser photocoagulation^16^, while in the case of TelCap that is very close to the fovea, a low laser power setting to avoid dense laser burn can be a factor for the low closure rate. Another possible cause is that we included only patients with DME refractory to anti-VEGF or corticosteroid injections, possibly reflecting the severity of the diffuse exudation.

Our study has several limitations. First, this was a retrospective and single-center study, which could have had a selection bias. Our study included patients with TelCaps refractory to anti-VEGF treatment, and these patients might not reflect general features of TelCaps that might have responded to anti-VEGF. This study also included a relatively small number of patients. Further, TelCap diagnosis was not based on ICGA. Instead, we evaluated whether the exudative TelCaps, which can be considered the cause of refractory edema, could be diagnosed using OCT and IR images. Therefore, the data presented in this paper might underestimate the presence of TelCaps. However, to the best of our knowledge, this study included the largest number of cases undergoing laser photocoagulation for TelCap reported thus far. IR and OCT image could not determine whether the leakage was exactly due to TelCap lesions. However, ICGA, the gold standard for TelCap diagnosis, was not routinely performed for DME in real-world practice. In addition, as shown in Fig. 1, it is likely that fluorescein angiography alone could not detect leaking TelCaps in chronic or persistent DME^10,13^. Taken together, our results support that IR imaging in conjunction with OCT can have a role in cases where ICGA is not available. In addition, OCT angiography has been used increasingly for managing DME^21^. Further research is needed to assess the agreement between IR and ICGA results and the efficacy of OCT angiography for TelCap diagnosis. Another limitation of this study was that it did not evaluate the recurrence of macular edema after one year. Well-designed prospective studies are needed to evaluate the long-term outcomes of TelCap laser photocoagulation.

In conclusion, IR imaging in conjunction with structural OCT may diagnose most of the TelCaps in persistent DME. The characteristic hyperreflectivity within the hyporeflective lesion on the IR image helps to identify TelCap. In addition, the results of this study suggest that IR and OCT-guided focal laser photocoagulation of TelCaps can improve functional and anatomical outcomes in patients with persistent DME.
